# Surface‐Spin‐Induced Magnetic Loss Enhancement in Ultralight Electromagnetic Absorbers

**DOI:** 10.1002/advs.76339

**Published:** 2026-06-26

**Authors:** Ruimin Ren, Ke Yang, Chichong Lu, Song Ma, Bowen Zheng, Zhenhui Ma

**Affiliations:** ^1^ Department of Chemistry School of Advanced Materials and Future Technology Beijing Technology and Business University Beijing China; ^2^ Department of Orthodontics, School and Hospital of Stomatology China Medical University Shenyang China; ^3^ Shenyang National Laboratory for Materials Science Institute of Metal Research Chinese Academy of Sciences Shenyang China

**Keywords:** electromagnetic wave absorption, Fe_3_O_4_, hollow structure, magnetic loss ability, surface atom spin

## Abstract

Limited by the intrinsic magnetic loss ability, the electromagnetic wave absorption (EMA) materials have encountered a bottleneck to achieve effective EMA with super‐low density. In this work, we propose a novel strategy to boost magnetic loss capacity by controlling surface atom spin. Using hollow Fe_3_O_4_ nanoparticles as a model, with their hollow rates controlled to 0, 17.1%, 32.2%, 46.6% and 54.1%, we reveal that the magnetic loss capacity is mainly dependent on the surface atom moment rather than the body moment. Attributed to the coexistence of inside and outside surface atoms moment, the 54.1% hollow Fe_3_O_4_ absorber represents about 20% enhancement in the imaginary part (*µ*'') than solid particles over 2–18 GHz. This improvement further makes the effective absorption bandwidth (≤‐5 dB) at 2.0 mm broaden to 11.91 GHz from 5.68 GHz in solid Fe_3_O_4_ nanoparticles and makes the absorption ability shift to low frequency. More importantly, the density of 54.1% hollow Fe_3_O_4_ absorber is only 48.9% of solid sample, which is very promising to meet the requirements for miniaturization and lightweighting. These findings provide important theoretical and practical guidelines for designing ultralight magnetic absorbers.

## Graphical abstract

Fe_3_O_4_ nanoparticles with different hollow rates were synthesized to demonstrate the key role of surface moment in magnetic loss process. Using the same volume filling ratio, the 54.1% hollow Fe_3_O_4_ absorber represents about 20% enhancement in the imaginary part (*μ*'') than solid particles due to the coexisting magnetic resonance of inside and outside surface atoms moment.

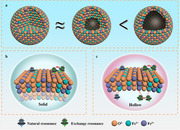

## 1 Introduction

The electromagnetic waves (EMWs) within the frequency range of 2–18 GHz have attracted widely attentions in mobile communication and radar detection fields [1, 2, 3, 4, 5, 6]. Correspondingly, the development of electromagnetic protection and radar stealth technologies has been an urgent demand [7, 8, 9, 10, 11]. The former can protect electronic component from the electromagnetic interference and make it operate well. And the latter can achieve the stealth feature of aircraft and weapon for defense applications. The emergence of EMW absorbers, which are composited of electromagnetic wave absorption (EMA) materials and organic binders, can address above issues effectively [1, 2, 12].

Current EMA materials mainly involve magnetic materials, carbon materials, as well as magnetic‐carbon composites, such as ferrites, graphene, carbon nanotube, ferrite/carbon hybrids, and so on [2, 4, 13, 14]. However, most of EMA materials mainly cover 8–18 GHz, and it is still a challenge to achieve the effective EMW attenuation within low frequency range (2–6 GHz), which is the key frequency band for 5G communication technology [1, 15, 16, 17].

Fundamentally, the absorbers attenuate EMW by conduction loss, dielectric loss and magnetic loss mechanisms [1, 2, 4, 5, 18, 19]. Hereinto, the magnetic loss mechanism exhibits the unique advance in attenuating EMW within low frequency range since the ferromagnetic resonance (usually natural resonance) generally occurs at 2–6 GHz. When an oscillating electromagnetic frequency matches the natural precession frequency of the magnetization, significant energy absorption and magnetization precession will emerge driven by internal magnetic anisotropy (magnetocrystalline or shape anisotropy) [1, 15, 16, 17, 20]. And this inherent resonance frequency *f*_R_ is determined by the anisotropy field (*H*_a_) and the gyromagnetic ratio (*γ*) [21]. Based on this theory, many magnetic materials, have been developed to achieve high performance EMA, such as Fe_3_O_4_, Fe based metals or alloys, Fe_4_N, and so on [22, 23, 24, 25]. However, these magnetic materials usually possess a magnetic loss limit due to their inherent characteristics, which hinders the improvement of performance. Furthermore, these magnetic absorbers exhibit a high density (>5 g cm^−3^ for ferrites, often 7–8 g cm^−3^ for Fe‐based metals/alloys) and require a large thickness (more than 5 mm) due to the limited magnetic loss ability, which is difficult to meet the requirements for miniaturization and lightweighting of the devices. Therefore, how to break through the magnetic loss limit and achieve an ultra‐low density have been the key challenge [1, 17, 26, 27].

To address above issues, in this work, we propose a novel magnetic loss model that can enhance magnetic loss ability and decrease absorber density dramatically. This model mainly involves the surface spin moment, instead of conventional body moment since the surface spin moment is easy to rotate under weak magnetic field due to the weak confinement by body atoms lattice energy [14, 15, 16, 28, 29]. Here, we selected Fe_3_O_4_ as the model material, owing to its high saturation magnetization (theoretical value ≈92 emu g^−1^), and its relatively low density (approximately 5.18 g cm^−^^3^). We first synthesized 500 nm Fe_3_O_4_ nanospheres with their hollow rate controlled at 0, 17.1%, 32.2%, 46.6% and 54.1%, respectively. Under the same volume filling ratio, the absorbers with hollow rate of 17.1%, 32.2% and 46.6% exhibit the same magnetic loss ability with solid Fe_3_O_4_ nanoparticles, while the 54.1% sample represents about 20% enhancement in the imaginary part (*µ*'') than solid particles over 2–18 GHz. The improvement of magnetic loss capacity further broadens the effective absorption bandwidth (≤‐5 dB) at 2.0 mm to 11.91 GHz from 5.68 GHz in solid Fe_3_O_4_ nanoparticles and makes the absorption ability shift to the low frequency. And the super‐low density of 2.005 g cm^−2^ is achieved in 54.1% Fe_3_O_4_ absorber, decreasing 51.1% than solid samples. The excellent EWA performances and super‐low density are attributed to the enhanced surface atom spin due to the coexistence of inside and outside surface atoms for 54.1% hollow Fe_3_O_4_ absorber. Our work proposes a novel magnetic loss mechanism, providing theoretical and practical guidelines for designing lightweight magnetic absorbers with tunable lowfrequency response.

## 2 Results and Discussion

**FIGURE 1:**
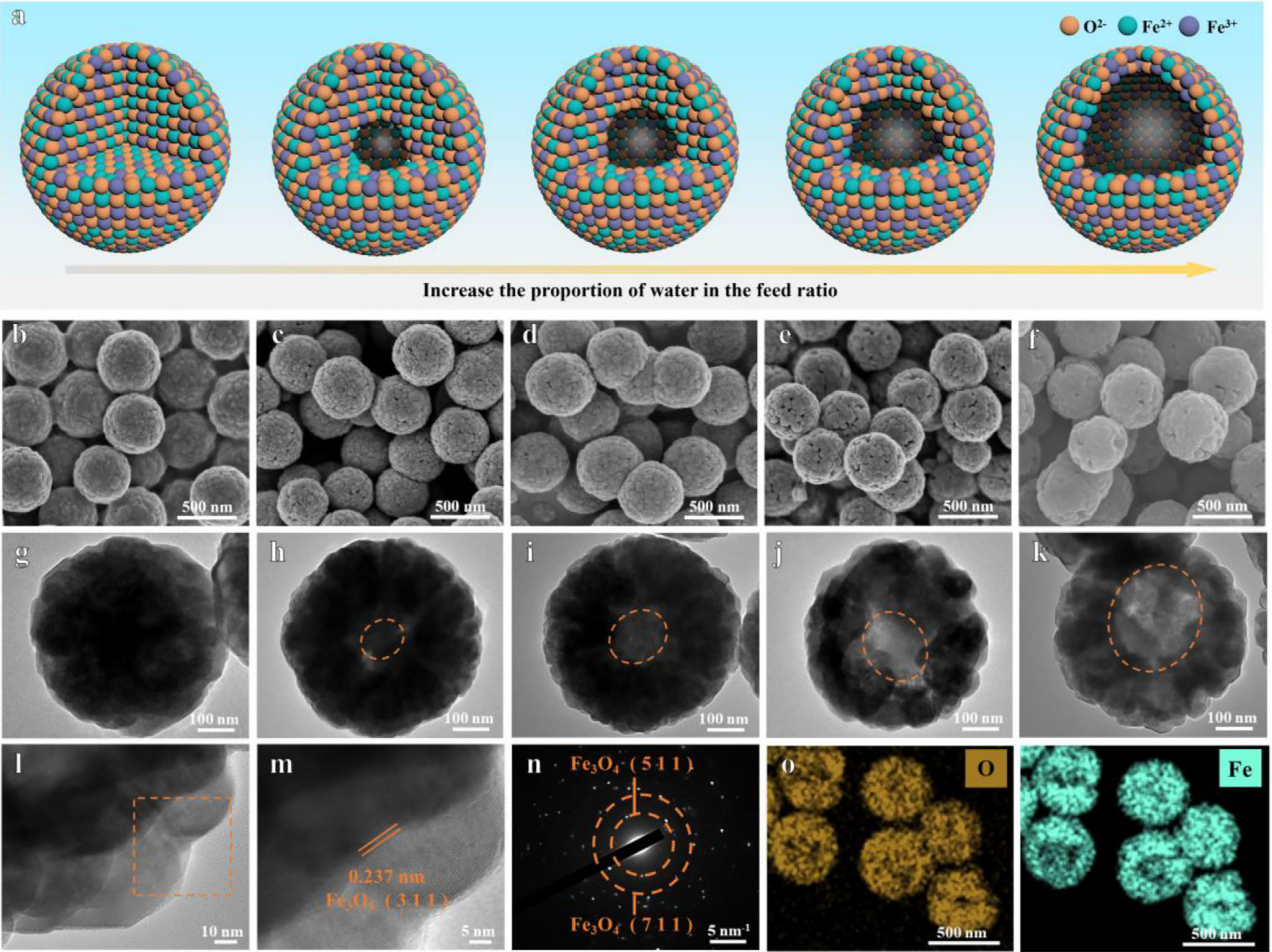
The synthetic process and microstructure characterization of Fe_3_O_4_ nanomaterials. a) The synthesis schematic diagram of hollow Fe_3_O_4_ nanomaterials. b–f) SEM images of samples with different hollow rate of b) 0.0%, c) 17.1%, d) 32.2%, e) 46.6%, f) 54.1%; g–k) TEM images of samples with different hollow rate of g) 0.0%, h) 17.1%, i) 32.2%, j) 46.6%, k) 54.1%; l–o) The characterizations of Fe_3_O_4_ nanoparticles with hollow rate of 54.1%, l) the magnified TEM image, m) HRTEM image, n) SAED pattern, o) the elemental mapping of Fe (green) and O (yellow).

Our synthesis started with the preparation of solid Fe_3_O_4_ nanospheres, as illustrated in Figure 1a. The solid Fe_3_O_4_ nanoparticles with an average diameter of 500 nm were obtained by reacting FeCl_3_ and H_2_O with Fe/H_2_O molar ratio of 1:6 in ethylene glycol solution using a hydrothermal technology at 200°C for 6 hours, as shown in Figure 1b (scanning electron microscopy, SEM). By controlling the Fe/H_2_O molar ratio, the hollow Fe_3_O_4_ nanospheres were successfully obtained without any change of particle size (Figure 1c–f) [30, 31]. And the ratio of 1:9, 1:12, 1:15, and 1:18 yield Fe_3_O_4_ nanoparticles with hollow rate of 17.1%, 32.2%, 46.6% and 54.1%, respectively, as observed in transmission electron microscopy (TEM) images (Figure g‐k). And the clear hollow structures have been marked as the orange dashed boxes. The high‐resolution TEM (HRTEM) of Fe_3_O_4_ nanoparticles with hollow rate of 54.1% (Figure 1m) displays clear lattice fringes with an interplanar spacing of 0.237 nm, which corresponds well to the (311) crystallographic planes of magnetite (Fe_3_O_4_). This phase assignment is further supported by the X‐ray diffraction (XRD) patterns of all samples, where the characteristic diffraction peaks match perfectly with the standard pattern for Fe_3_O_4_ (PDF card: 79–0419) (Figure S1). And the selected area electron diffraction (SAED) pattern (Figure 1n) shows the distinct diffraction rings, suggesting the polycrystal structure of hollow Fe_3_O_4_ nanoparticles. The elemental mapping results (Figure 1o and Figure S2), collectively confirm the homogeneous distribution of Fe and O elements throughout these nanospheres.

**FIGURE 2:**
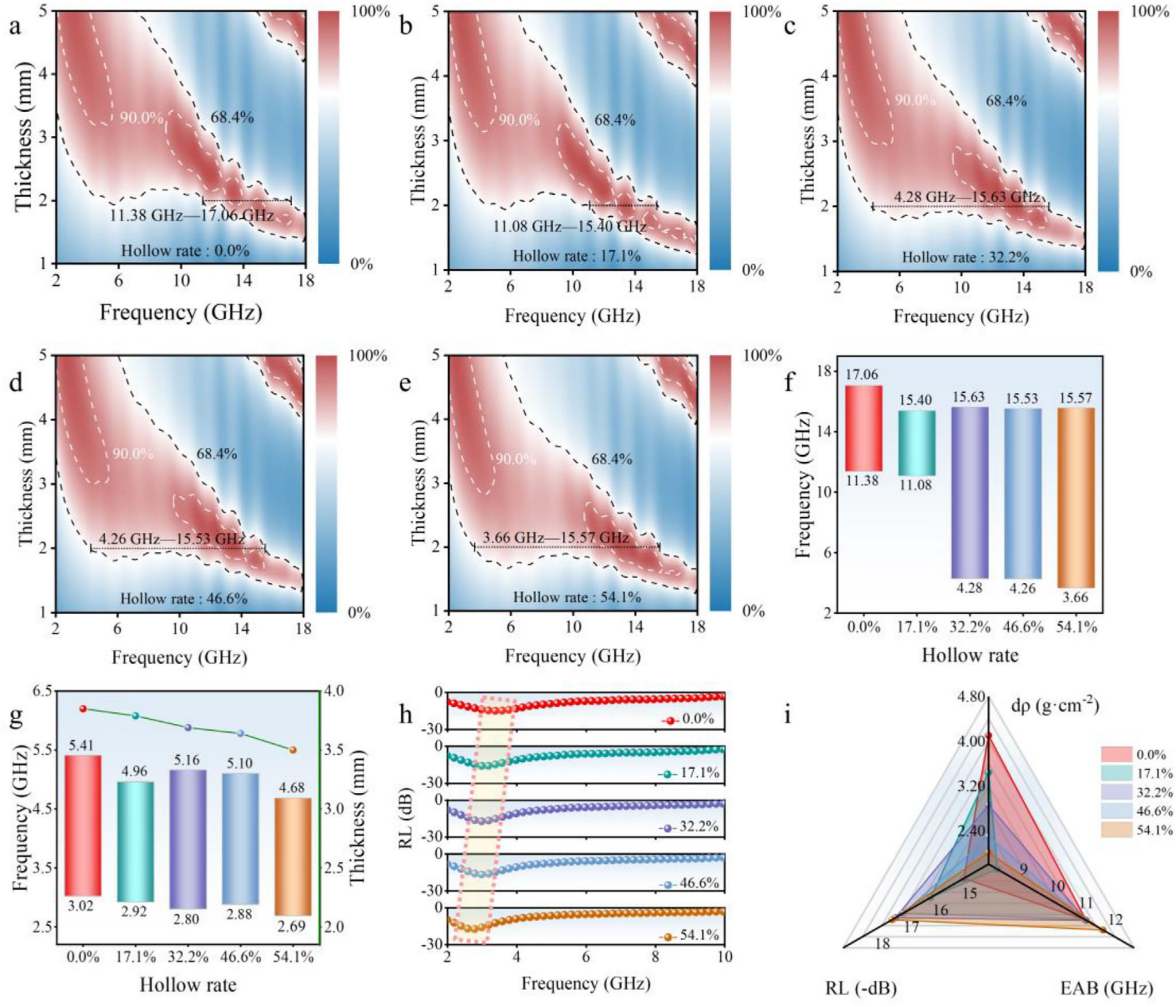
The EMA performances of Fe_3_O_4_ samples with different hollow rates: a–e) *η* maps of samples with hollow rates of 0.0%, 17.1%, 32.2%, 46.6%, and 54.1%; f) EAB ranges (RL ≤ −5 dB) at 2.0 mm for Fe_3_O_4_ samples with hollow rates of 0.0%, 17.1%, 32.2%, 46.6%, and 54.1%. g) The EAB_max_ values (RL ≤ −10 dB) in the 2–6 GHz band and their corresponding matching thicknesses for Fe_3_O_4_ samples with hollow rates of 0.0%, 17.1%, 32.2%, 46.6%, and 54.1%; h) The RL values within 2–10 GHz at 4.5 mm. i) The comprehensive comparison chart of area density (*d*ρ) –EAB (≤‐5 dB)– RL_min_ for all samples.

These as‐synthesized Fe_3_O_4_ nanoparticles were further mixed with paraffin to prepare absorbers and measure their absorption performance. Using the same volume filling ratio (75%), meaning the same nanoparticles number and different weight, the EMW absorption performances of Fe_3_O_4_ nanoparticles with hollow rates of 0.0%, 17.1%, 32.2%, 46.6% and 54.1% were evaluated by absorption efficiency (*η*) and reflection loss (RL), as shown in the corresponding thickness‐dependent 3D RL curves and their corresponding RL–frequency curves (Figures S3–S7). In detail, Figure 2a–e map *η* as a function of frequency and thickness, where the *η* = 90.0% and *η* = 68.4% contours are highlighted, corresponding to RL ≤ −10 dB (90.0% absorption efficiency) and RL ≤ −5 dB (68.4% absorption efficiency) [32, 33], respectively, since the assessment criteria is usually fixed to RL ≤ −5 dB in practical aircraft and weapon applications. At a matching thickness of 2.0 mm, the effective absorption bandwidth (EAB with RL≤−5 dB) is 5.68, 4.32, 11.35, 11.27 and 11.91 GHz, for Fe_3_O_4_ nanoparticles with hollow rate of 0.0%, 17.1%, 32.2%, 46.6% and 54.1%, respectively. The expanded EAB is mainly originated from the enhanced absorption at low frequency for high hollow rate samples, suggesting that the hollowing particles can facilitate both absorption ability and low density. Figure 2f further summarizes the bandwidth variation at *η* = 68.4% (RL ≤ −5 dB) under the matching thickness of 2.0 mm, where the EAB is progressively broadened and shifts to low frequency with increasing hollow rate, with the 54.1% sample exhibiting the widest bandwidth. The EAB_max_ (RL≤ −−10 dB) is summarized as Figure 2g, which also demonstrates that the EAB_max_ value shifts to low frequency and the thickness is decreasing with the increase of hollow rate. Similar phenomenon can be observed in RL_min_ values (Figure 2h) at the thickness of 4.5 mm, where RL_min_ values reduce (from −14.85 to −17.32 dB) and shift to low frequency (from 3.3 to 2.7 GHz). Figure 2i gives a comprehensive comparison chart of area density (dρ), EAB and RL_min_ for Fe_3_O_4_ nanoparticles with different hollow rate, where 54.1% sample exhibits the lowest dρ of 2.005 g cm^−2^, only 48.9% of solid sample. The super low area density, relatively strong absorption ability (RL_min_ = −17.32 dB), as well as a wide EAB (from 3.66 to15.57 GHz), confirming the promising applications in high‐performance EMA materials with the lightweight feature [7, 23, 31].

The absorption performance of the 54.1% hollow Fe_3_O_4_ absorber was further compared with representative hollow ferrite, carbon‐based and MXene‐based absorbers, as summarized in Tables S1 and S2. Under the same criterion of RL ≤ −10 dB, corresponding to ≥ 90% absorption efficiency, our hollow Fe_3_O_4_ absorber shows a low‐frequency effective absorption band of 2.69–4.68 GHz at a matching thickness of 3.50 mm, demonstrating its advantage in low‐frequency absorption with a relatively thin thickness.

**FIGURE 3:**
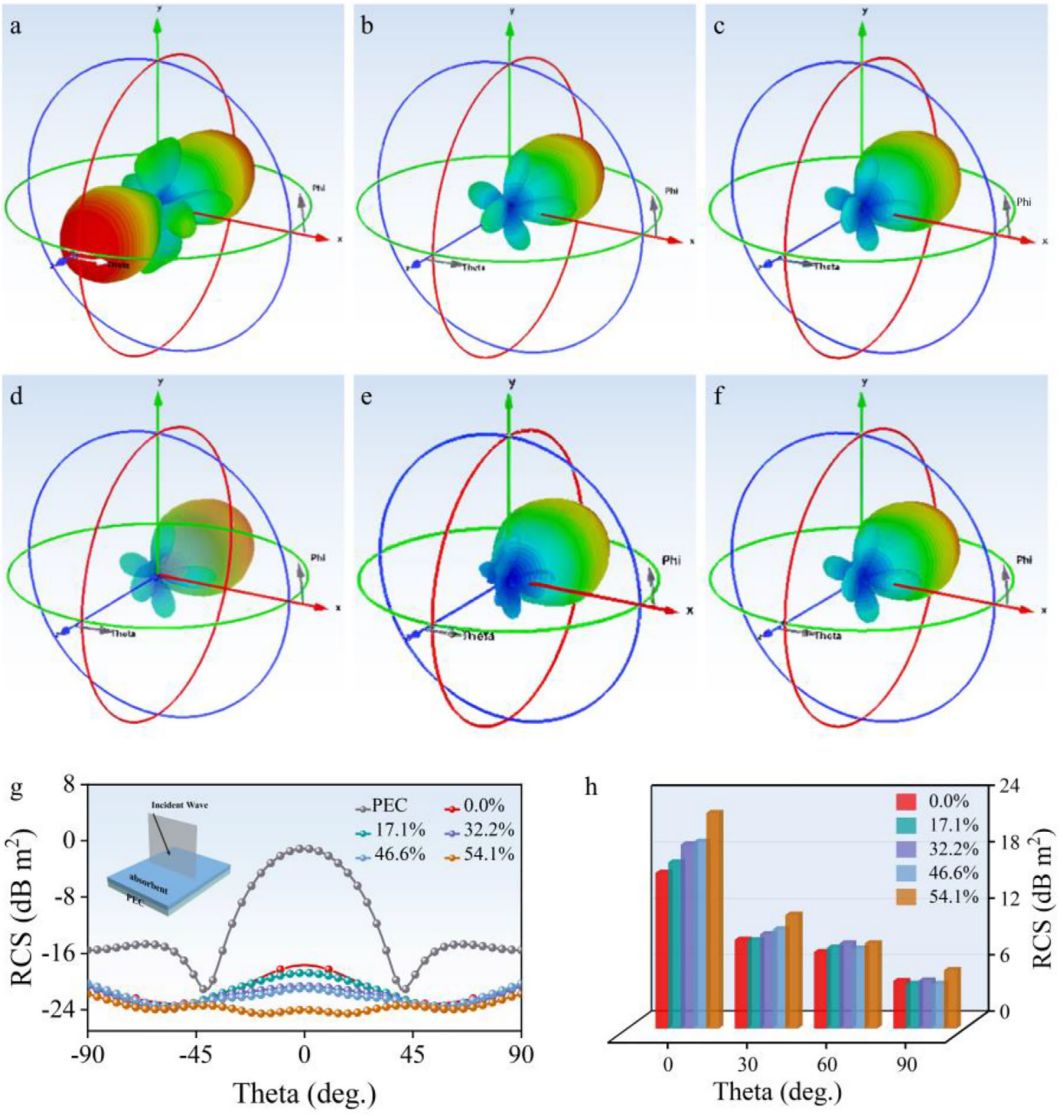
The RCS simulation results of Fe_3_O_4_ nanoparticles. a–f) 3D RCS plots. g) RCS simulation curves for all the Fe_3_O_4_ nanoparticles and PEC at scanning angles of ‐90° to 90 °. h) RCS reduction values.

To further assess the practical application potential of the synthesized Fe_3_O_4_ nanoparticles, radar cross‐section (RCS) simulations were performed using CST Studio Suite [34, 35, 36]. The simulation model features a perfect electric conductor (PEC) substrate coated with the Fe_3_O_4_ nanoparticles, with a plane wave incident along the negative z‐−axis. Figure 3a displays the 3D RCS scattering signal distribution for the bare PEC substrate. And the corresponding results for substrates coated with Fe_3_O_4_ nanoparticles with hollow rates of 0.0%, 17.1%, 32.2%, 46.6%, and 54.1% are shown in Figure 3b–f. Compared to the bare PEC substrate, the samples coated with Fe_3_O_4_ nanoparticles show significantly reduced scattering signals in the 3D RCS patterns, indicating a substantial improvement in attenuation performance. This observation confirms the superior EMW dissipation capability of all samples. Furthermore, 2D RCS simulation curves at different angles are provided in Figure 3g,h. These results indicate that the Fe_3_O_4_ nanoparticles with 54.1% hollowness achieve an RCS reduction of 22.89 dB·m^2^ at 0°, suggesting the optimal microwave attenuation characteristics. Notably, across the entire scanning angle range (−90° to 90°), the RCS values for all Fe_3_O_4_ samples with varying hollow rates (0.0%, 17.1%, 32.2%, 46.6%, and 54.1%) remain below −15 dB·m^2^. This consistent performance further supports their strong potential for practical application in tackling electromagnetic wave dissipation challenges, especially in scenarios requiring broad‐angle coverage.

**FIGURE 4:**
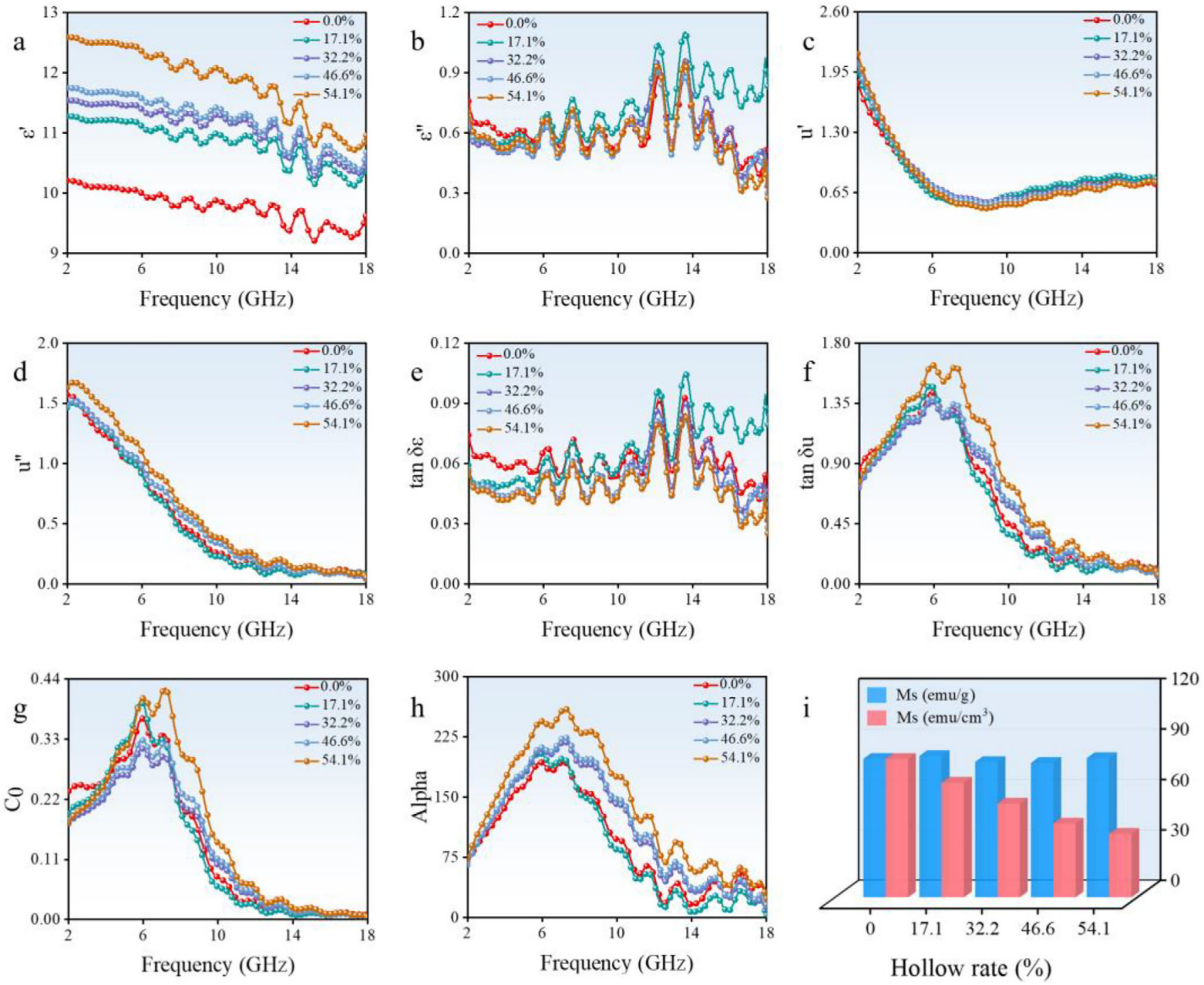
The electromagnetic parameters of Fe_3_O_4_ samples with different hollow rates. (a) ε' values, (b) ε“ values, (c) µ' values, (d) *µ*” values, (e) tan*δ*_ε_ values, (f) tan*δ*_µ_ values, (g) *C*_0_ values, (h) α values, (i) Saturation magnetization (gravimetric and volumetric values).

To investigate the EMA mechanisms, the electromagnetic parameters of Fe_3_O_4_ samples with varying hollowness (0%, 17.1%, 32.2%, 46.6%, 54.1%) over the frequency range of 2–18 GHz have been given in Figure 4. The *ε*' value generally represents the ability to store electrical energy. And the significant enhancement of *ε*' value with the increase of hollow rate (Figure 4a), is mainly attributed to the “micro‐capacitors” effect. In our system, a hollow Fe_3_O_4_ nanoparticle can be regarded as a micro‐capacitor. And with the increase of hollow rate, the capacitance of Fe_3_O_4_ nanoparticles will correspondingly enhance. And thus, the *ε*' values increase monotonously with the increase of Fe_3_O_4_ nanoparticles hollow rate [14, 37, 38, 39]. The *ε*'' which demonstrates the material's ability to dissipate electrical energy, exhibit the similar values (Figure 4b), suggesting the hollow structure has little effect on dielectric loss ability [40]. The dielectric loss ability is dependent on the polarization relaxation, involving interface polarization, dipole polarization, defect polarization, as so on [41]. Generally, the interface polarization can response EMW at 6–8 GHz, dipole polarization originated from particles occurs at 12–14 GHz, and the defect polarization corresponds to the resonance peaks around 18 GHz [19]. In our system, the Fe_3_O_4_ nanoparticles with different hollow rate have similar interface polarization, dipole polarization and defect polarization. As a result, they exhibit the close dielectric loss ability. Similar phenomenon can be found in the real part (*µ*') of the complex permeability, where all sample represent the close *µ*' values, as shown in Figure 4c. Different from the change trend of *µ*' value, the imaginary part (*µ*'') is increasing with the enlarged hollow rate over frequency range (2–18 GHz). Especially, for the Fe_3_O_4_ nanoparticles with hollow rate of 54.1%, they exhibit obvious enhancement in *µ''* value. It can be attributed to the enhanced interaction between surface atoms spin and EMW since the high hollow rate can provide double surface atoms [30, 42].

The dielectric loss tangent value (tan*δ*_ε_) and the magnetic loss tangent value (tan*δ*_µ_) have been shown in Figure 4e,f, respectively. Compared with tan*δ*_ε_, the tan*δ*_µ_ value are obviously higher at 2–18 GHz, suggesting that the magnetic loss mechanism plays a dominant role in this EMA system. The corresponding *C*_0_ values were shown in Figure 4g. The relatively high *C*_0_ values have been obtained within 2–8 GHz for all samples, suggesting that the natural resonance (2–6 GHz) and exchange resonance (6‐14 GHz) play a dominant role in magnetic loss process. Whereas, the *C*_0_ value at high frequency represents a constant close to 0, which indicates that there is almost no eddy current loss in this system due to their insulation features [43, 44, 45]. The attenuation constant (*α*) is further used to evaluate the inherent capability to dissipate electromagnetic energy [35]. As depicted in Figure 4h, the *α* value increases significantly with the hollow rate, rising from around 190 for the solid sample to approximately 250 for the sample with 54.1% hollowness. The enhancement of α value is mainly originated from the improved magnetic loss ability induced by the surface atoms spin of hollow nanoparticles. It gives a direct evidence that the hollow structure substantially improves the intrinsic attenuation capacity over 2–18 GHz range. Figure S8 shows the magnetic hysteresis loops of Fe_3_O_4_ samples with different hollow rates. All samples exhibit typical soft magnetic behaviors with high saturation magnetization and small coercivity. The gravimetric saturation magnetization values (*M*_s_) are close for all samples (around 82 emu g^−1^), indicating that the hollow structure does not significantly change the intrinsic magnetic moment of Fe_3_O_4_. Meanwhile, the coercivity values show only slight variation (around 75 Oe) without a clear monotonic dependence on the hollow rate. It demonstrates that similar Fe_3_O_4_ phase composition and comparable particle size (close defects) lead to the close magnetic properties. In this work, the electromagnetic parameters were obtained using the same volume filling ratio, and thus the volumetric *M*_s_ (emu cm^−3^) is very meaningful. Figure 4i compares the saturation magnetization (*M*_s_) of the samples with different hollow rates in both gravimetric (emu g^−1^) and volumetric (emu cm^−3^) forms. The decrease of volumetric *M*_s_ with increasing hollow rate is attributed to the removal of the magnetic core. After removing the magnetic core, these samples exhibit close *µ*' and *µ*'' values for the Fe_3_O_4_ samples with hollow rate of 0.0%, 17.1%, 32.2% and 46.6%, demonstrating that the surface moment of Fe_3_O_4_ play a key role rather than the body moment. It can be explained that the magnetic moments on the surface are easier to interact with EMW than bulk moments due to the weak limitation of surface magnetocrystalline anisotropy. For 54.1% hollow sample, it shows enhanced magnetic loss capability (higher *µ*'' values) than other samples, which should be attributed to the inner surface moment. When the hollow rate is lower than 54.1%, the EMW is difficult to penetrate Fe_3_O_4_ shell, and only the outer surface moments interact with EMW. When the hollow rate reaches 54.1%, the EMW is easy to penetrate Fe_3_O_4_ shell (≈50 nm thickness), and both the outer and inner surface moment interact with EMW. Therefore, in fact, the magnetic loss ability is mainly dependent on the number of magnetic moments that interacts with EMW. The more numerous, the stronger the loss capacity.

The electron spin resonance (ESR) measurements were performed to further probe the spin states of solid and 54.1% hollow Fe_3_O_4_. As shown in Figure S9a, both the solid and 54.1% hollow Fe_3_O_4_ samples show resonance signals near *g* = 2.003, indicating that the active spin centers are mainly originating from oxygen vacancies. However, the 54.1% hollow sample exhibits a larger peak‐to‐peak linewidth Δ*H*_pp_ of 1.56 G than 1.27 G of the solid sample (Figure S9b). The increased linewidth suggests more oxygen vacancies in the hollow structure than solid samples, which is mainly from the increased oxygen vacancies on the inner shell. These results indicate that the 54.1% hollow Fe_3_O_4_ nanoparticles have more active inner/outer surface spins participating in electromagnetic‐field‐induced spin relaxation and resonance than other samples, leading to the enhanced magnetic loss ability.

The COMSOL Multiphysics simulations were further conducted to confirm that EMWs can reach the inner surface of hollow Fe_3_O_4_ particles. The corresponding geometric models and electromagnetic loss‐density distributions at 10 GHz have been shown in Figures S10 and S11. The 54.1% hollow particle with an approximately 50 nm shell exhibits obvious loss density across the thin shell and near the inner surface at 10 GHz, indicating that EMWs can penetrate the shell. This result supports the participation of inner‐surface spin moments in EMW‐induced magnetic loss, thereby explaining the enhanced *µ''* and attenuation constant *α* of the 54.1% hollow Fe_3_O_4_ sample.

**FIGURE 5:**
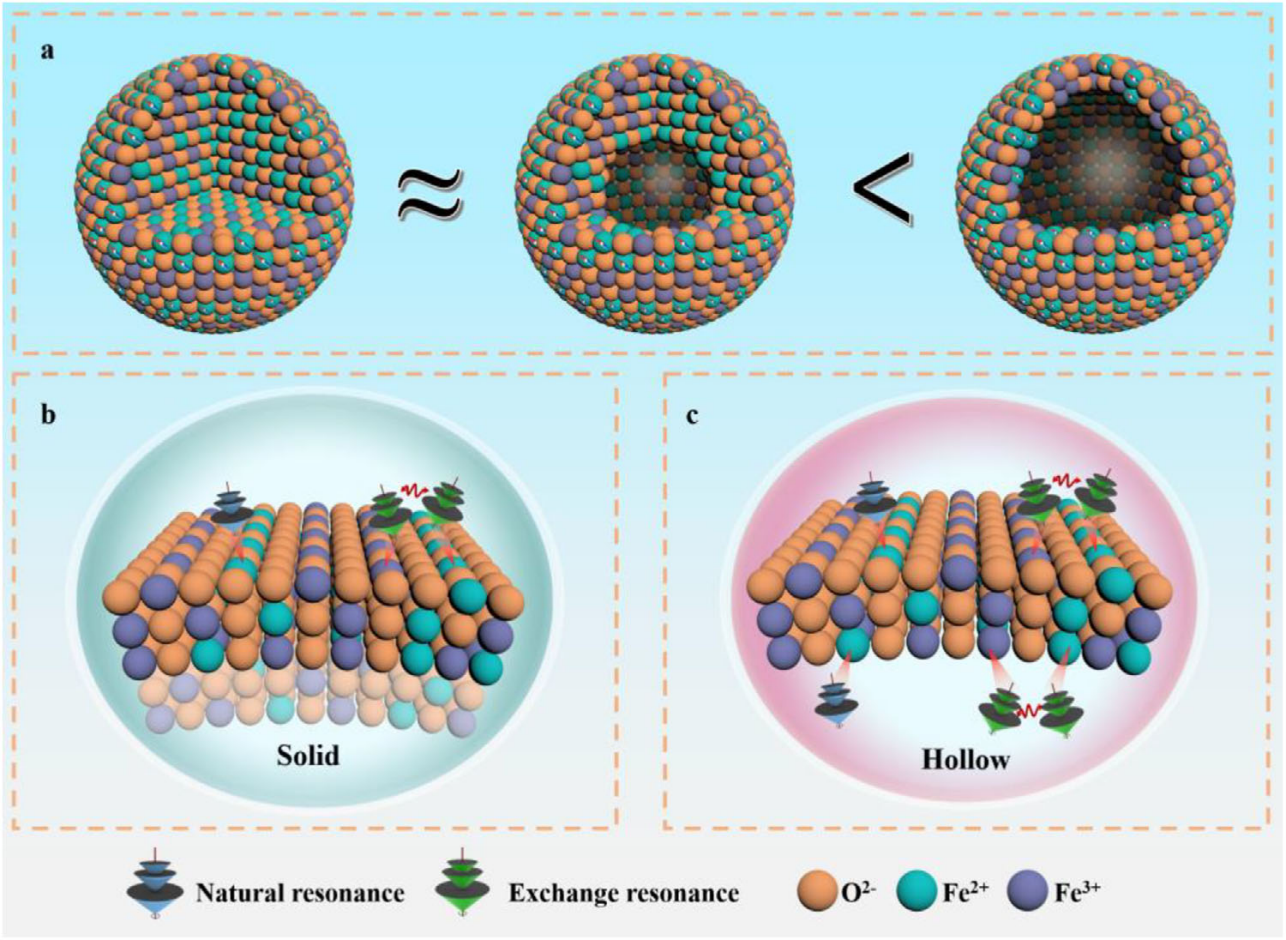
The mechanism for electromagnetic dissipation in this system.

The EMA mechanisms have been further summarized in Figure 5. For Fe_3_O_4_ nanoparticles with hollow rates of 17.1%, 32.2% and 46.6%, they exhibit similar surface spin moments with solid spheres, while the Fe_3_O_4_ nanoparticles with hollow rate of 54.1% show more surface spin moments due to the existence of internal surface and external surface atom moments (Figure 5a). When the EMW enter absorbers, they can penetrate the surface of Fe_3_O_4_ hollow sphere and thus induce the rotation of internal surface moments due to the super thin shell (≈50 nm) for Fe_3_O_4_ particles with hollow rate of 54.1%. As a result, the samples with hollow rate of 54.1% exhibit the highest magnetic loss ability even with the lowest density. For solid particles or the hollow particles with thick shells, the internal atoms are bonded by lattice energy and the corresponding spin moment is difficult to rotate under EMW radiation (Figure 5b). For Fe_3_O_4_ particles with hollow rate of 54.1%, the shell thickness of ≈50 nm can allow EMW enter the core of particles and induce the atom moment rotation on inner shell (Figure 5c). As a result, the Fe_3_O_4_ particles with hollow rate of 54.1% exhibit obviously enhanced magnetic loss ability than other samples.

Within low frequency range of 2–6 GHz, the natural resonance is the main magnetic loss source, where atoms spin moments contribute to all the natural resonance. The natural resonance arises from the collective precession of magnetic moments, which is primarily governed by magnetocrystalline anisotropy field. Resonance and magnetic loss occur when the frequency of the EMW matches the precession frequency of magnetic moments. Within 6–14 GHz, the exchange resonance originated from inhomogeneous precession play a key role. In ferromagnetic materials, atomic magnetic moments are bound by exchange interactions. Under the excitation of an external electromagnetic field, these precession of magnetic moments at different positions generates a phase difference and forms a spin wave. When the frequency of the external electromagnetic wave coincides with the eigenfrequency of this spin wave, resonance occurs, and energy is absorbed and dissipated. For metal ferromagnets, the exchange interaction arises the direct exchange effect between neighboring metal atoms. For ferrites, the exchange interaction arises the super‐exchange interaction such as Fe‐O‐Fe. The exchange resonance can be regarded as the natural resonance extension caused by the inhomogeneous rotation of magnetic moments and it is expressed as Equation (1).

(1) $$f_{e}=f_{n}+\frac{1}{2\pi }\times \gamma \left(\frac{2A\mu_{kn}^{2}}{r^{2}M_{s}}\right)$$

where *A* is the exchange constant, *µ*_kn_ is the eigenvalue of the derivative of the spherical Bessel function, and the *r* is the radius of particles [46, 47]. And thus, a high exchange constant will lead to a high exchange resonance frequency. And the resonance linewidths are related with the radius of particles. A narrow size distribution will cause narrow resonance linewidth. In our work, the size of Fe_3_O_4_ is well‐distribution, and thus the resonance linewidths of all samples are narrow.

These results prove that the magnetic core of body materials has no contribute to EMW attenuation, and the surface spin moment play a key role in the magnetic loss process [5, 6, 18, 23, 30, 42].

## 3 Conclusion

In summary, we successfully synthesized 500 nm Fe_3_O_4_ nanoparticles with their hollow rate controlled at 0.0%, 17.1%, 32.2%, 46.6% and 54.1%. Under the same volume filling ratio, the 54.1% samples exhibit excellent EMW absorption performances with EAB_max_ (RL≤−5 dB) of 11.9 GHz (from 3.66 to 15.57 GHz) at 2.0 mm and super‐low density of 2.005 g cm^−2^, demonstrating the promising applications to meet the requirements for miniaturization and lightweighting of the devices. The enhanced EMA ability is mainly attributed to the increase of surface atom spin moment since the EMW can induce double surface atom spin (on internal surface and external surface) due to the thin shell for 54.1% Fe_3_O_4_ nanoparticles. Certainly, the hollow structure also significantly reduces the density of absorbers to achieve the lightweight. Using this model, we prove that the magnetic core of body materials has no contribute to EMW attenuation, and the surface spin moment plays a key role in the magnetic loss process, which provides an important researching thought to understand the EMW loss mechanism and design the high‐performance absorbers.

## Author Contributions

**Ruimin Ren** and **Ke Yang** did investigation and performed formal analysis. Ruimin Ren wrote the original draft. **Song Ma** contributed to review and editing. **Zhenhui Ma**, **Chichong Lu**, and **Bowen Zheng** contributed to conceptualization, writing, review and editing, and project administration (equal).

## Conflicts of Interest

The authors declare no conflicts of interest.

## Supporting information




**Supporting File**: advs76339‐sup‐0001‐SuppMat.docx.

## Data Availability

The data that support the findings of this study are available from the corresponding author upon reasonable request.
