# Supplementary material for: Surface‐Spin‐Induced Magnetic Loss Enhancement in Ultralight Electromagnetic Absorbers
Source: Adv Sci (Weinh). 2026 Jun 26:e76339. Online ahead of print. doi: 10.1002/advs.76339 (PMC13336841; doi:10.1002/advs.76339)
Supplement: Supplementary file 1 — Supporting File: advs76339‐sup‐0001‐SuppMat.docx. [file ADVS-9999-e76339-s001.docx]

**Supporting Information**

**Surface-spin-induced magnetic loss enhancement in ultralight electromagnetic absorbers**

Ruimin Ren^1#^, Ke Yang^1,2#^, Chichong Lu^1^*, Song Ma^3^, Bowen Zheng^2^*, Zhenhui Ma^1^*

1. *School of Advanced Materials and Future Technology,* *Beijing Technology and Business University, Beijing 100048, China*

2. *Department of Orthodontics, School and Hospital of Stomatology, China Medical University, Shenyang, 110002, China*

3. *Shenyang National Laboratory for Materials Science, Institute of Metal Research, Chinese Academy of Sciences, Shenyang, 110016, China*

# R. Ren and K. Yang contributed equally.

* Corresponding authors: luchichong@btbu.edu.cn (C. Lu); bwzheng@cmu.edu.cn (B. Zheng); mazh@btbu.edu.cn (Z. Ma).

**1. Experimental Section**

**The synthesis of Fe_3_O_4_ nanoparticles**

Fe_3_O_4_ nanoparticles were synthesized via a solvothermal method in ethylene glycol (EG). Typically, FeCl_3_ (AR, Mw = 162.2, Aladdin) (2.70 g, 16.65 mmol) was dissolved in EG (≥98%, Mw = 62.07, Aladdin) (10 mL) under magnetic stirring (500 rpm) at room temperature. To regulate the hollowness of the products, deionized water was added to the FeCl_3_/EG solution (V(H_2_O) = 1.80, 2.70, 3.60, 4.50, and 5.40 mL), corresponding to molar ratios of n(Fe³⁺):n(H_2_O _added) = 1:6, 1:9, 1:12, 1:15, and 1:18, respectively. Subsequently, polyethylene glycol (PEG, Mn = 2000, PEG-2000, Aladdin) (0.75 g) was added and stirred until complete dissolution. In parallel, sodium acetate (NaAc, AR, Mw = 82.03, Aladdin) (2.87 g) was dissolved in EG (20 mL) under magnetic stirring (500 rpm). The FeCl_3_-containing solution was introduced dropwise into the NaAc solution at a rate of 5 mL·min⁻¹ using a syringe pump under stirring (500 rpm). After the addition, the mixture was further stirred for 30 min to ensure homogeneity and then transferred into a 50 mL Teflon-lined stainless-steel autoclave (filling ratio ~64–71%). The autoclave was heated at 200 °C for 6 h and naturally cooled to room temperature. The resulting black precipitate was collected by centrifugation (8000 rpm, 8 min), washed with deionized water and ethanol (three times each, ~15 mL per wash), and dried in an air oven at 60 °C for 6 h to obtain Fe_3_O_4_ powder.

**2. Characterizations**

The crystal structure was investigated by X-ray diffraction (XRD, Bruker D8 Advance) analysis with the scanning rate to 5° min^-1^. The element distributions were determined by energy dispersive spectroscopy (EDS). The morphologies and microstructures of Fe_3_O_4_ were studied by scanning electron microscopy (SEM, S–4800) and transmission electron microscopy (TEM, FEI Tecnai G2 F20). The ratios of element were determined by elemental analysis using a JY2000 Ultrace inductively coupled plasma atomic emission spectrometer. Magnetic properties of the Fe_3_O_4_ were collected by Vibrating Sample Magnetometer (VSM, Versalab) at 300 K at the external field (H) ranged from -25 to 25 kOe. The electron paramagnetic resonance (EPR) spectra were recorded using an EMX A300 EPR spectrometer (Bruker, Germany) at room temperature.

The measurement of electromagnetic parameters was based on a vector network analyzer (VNA, Agilent E5071C). Typically, the Fe_3_O_4_ were mixed with a 75% volume ratio of paraffin wax matrix in a 10 ml beaker, followed by the addition of 2 ml of n-hexane. Under vigorously stirring, the mixture was heated to 80°C to melt the paraffin completely. After removing n-hexane, the system was cooled down to room temperature and obtain a solid mixture, which was placed in a coaxial mold with an inner diameter of 3.04 mm and an outer diameter of 7 mm. After pressing under a pressure of 2,000,000 Pa, the sample is molded and then demolded to obtain a coaxial ring sample with a thickness of approximately 2 mm.

The RL was calculated by the following formula based on the transmission line theory:

$RL=20{log}_{10}\left| \frac{\left( Z_{in}-Z_{0} \right)}{Z_{in}+Z_{0}} \right|$ (Equ.S1)

$Z_{in}=Z_{0}\sqrt{\frac{{}_{r}}{\varepsilon_{r}}}\tanh\left( j\frac{2\pi fd}{c}\sqrt{{}_{r}\varepsilon_{r}} \right)$ (Equ.S2)

In the formula, Z_in_ and Z_0_ represent the input impedance of the absorber and the air; d is the thickness and of the absorber. ƒ and c are the frequency of the EMW and the speed of light in vacuum, respectively. Impedance matching (|Z_in_/Z_0_|) is ratio of the input impedance to the free-space impedance, which is commonly used to assess the extent to which the incident EM wave enters the absorber. Impedance matching values close to 1 suggest that the incident EM wave almost completely into the material rather than reflecting at the surface.

The single-particle electromagnetic simulations were performed using COMSOL Multiphysics. Three-dimensional Fe_3_O_4_ spherical models with a fixed outer diameter of 500 nm were constructed, and the inner cavity dimensions were adjusted according to the experimentally determined hollow rates of 0.0%, 17.1%, 32.2%, 46.6%, and 54.1%. The particles were placed in an air domain, and the experimentally measured complex permittivity and permeability of the corresponding samples at 10 GHz were assigned as the material parameters. A linearly polarized plane electromagnetic wave was incident on the particles, and scattering boundary conditions were applied to the outer boundaries of the computational domain to simulate free-space propagation. The mesh was refined at the Fe_3_O_4_ shell and the inner and outer interfaces. The same excitation conditions, boundary settings, and meshing criteria were used for all models. The electromagnetic loss-density distributions at 10 GHz were extracted from the central cross-sections of the particles to evaluate the electromagnetic energy dissipation and the interaction of the incident wave with the inner and outer surfaces of the hollow Fe_3_O_4_ particles.

Radar equation and radar cross section (RCS) simulation theory further confirming EM wave absorption performance. RCS simulation was obtained by the following formula:

$\sigma\left( dBm^{2} \right)=10log\left( 4\pi\frac{S}{\lambda^{2}} \frac{\left| E_{s} \right|^{2}}{\left| E_{i} \right|^{2}} \right)$ (Equ.S3)

where S, λ, E_s_ and E_i_ are the area of the simulated plate, the wavelength of the incident EMW, the scattered field intensity of the transmitted waves, and the incident field intensity of the received waves, respectively.


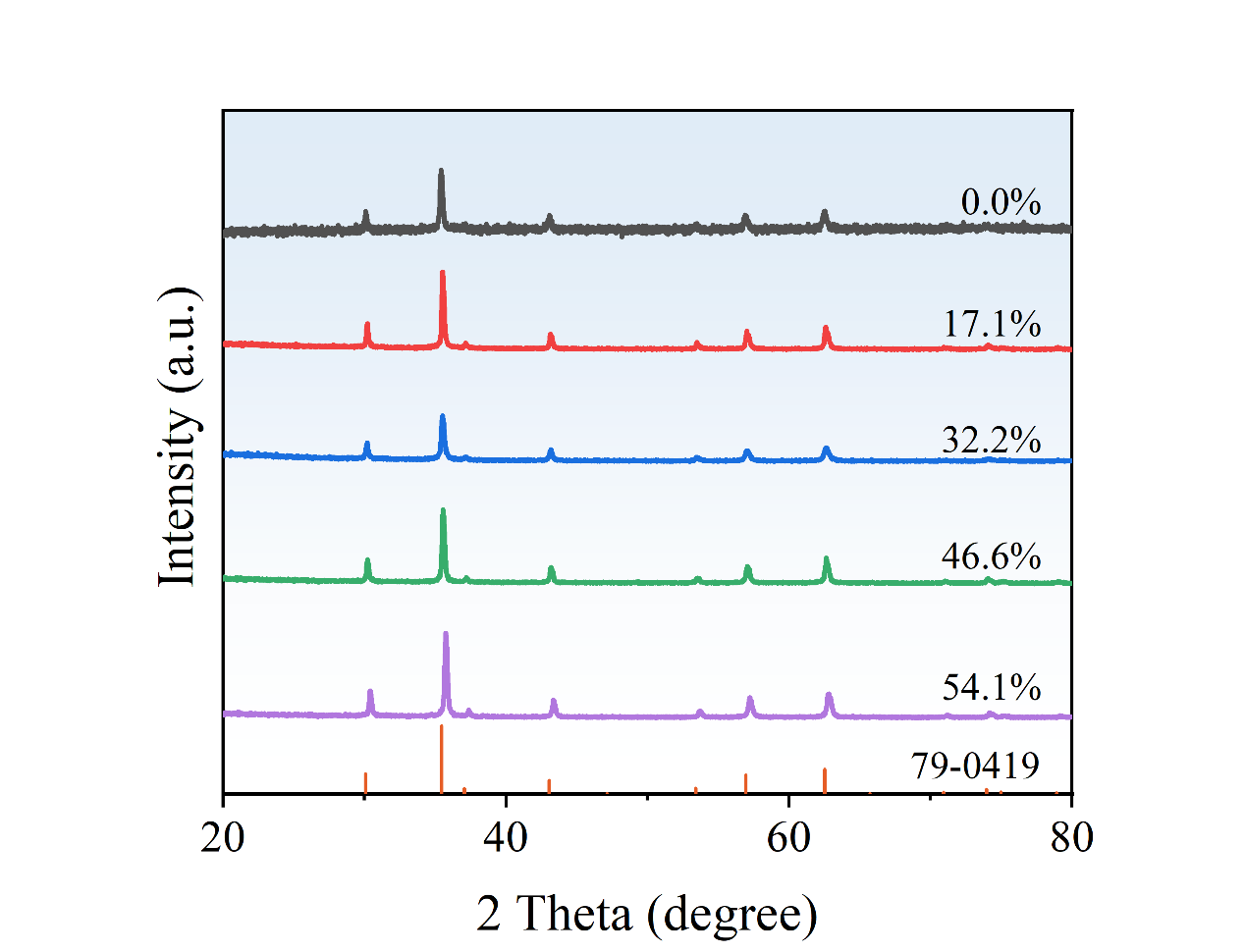


Figure S1 XRD patterns of Fe_3_O_4_ samples with different hollow ratios (0.0%, 17.1%, 32.2%, 46.6% and 54.1%).


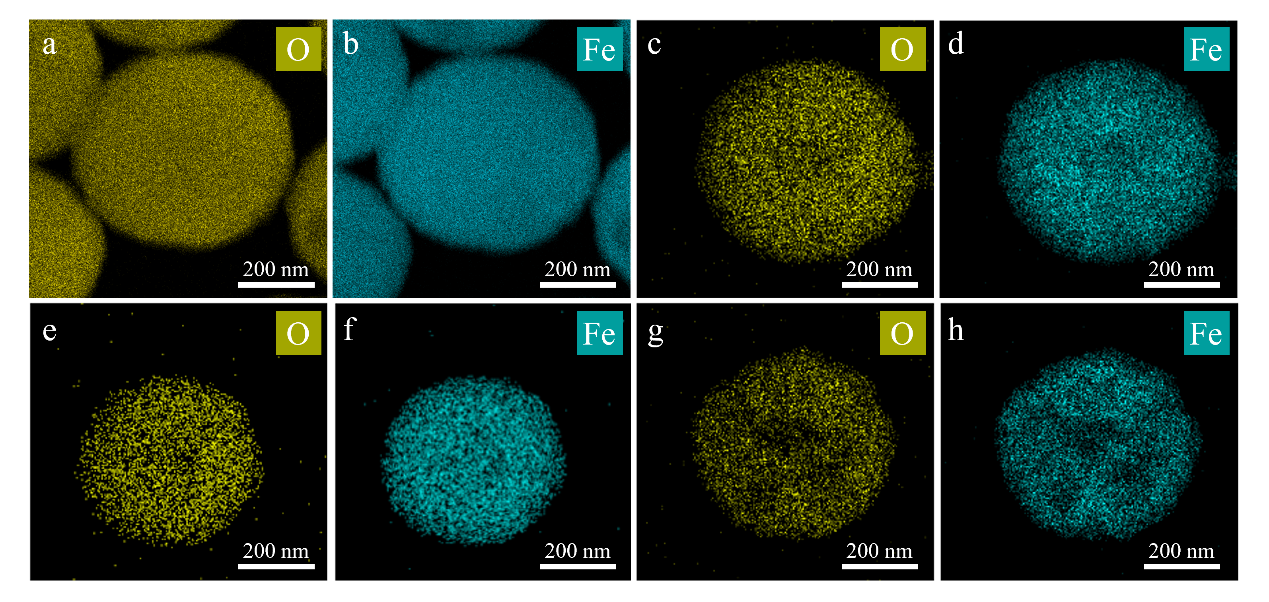


Figure S2 EDS elemental mapping images of O and Fe for Fe_3_O_4_ microspheres with different hollow ratios. (a, b) 0.0%, (c, d) 17.1%, (e, f) 32.2% and (g, h) 46.6% .


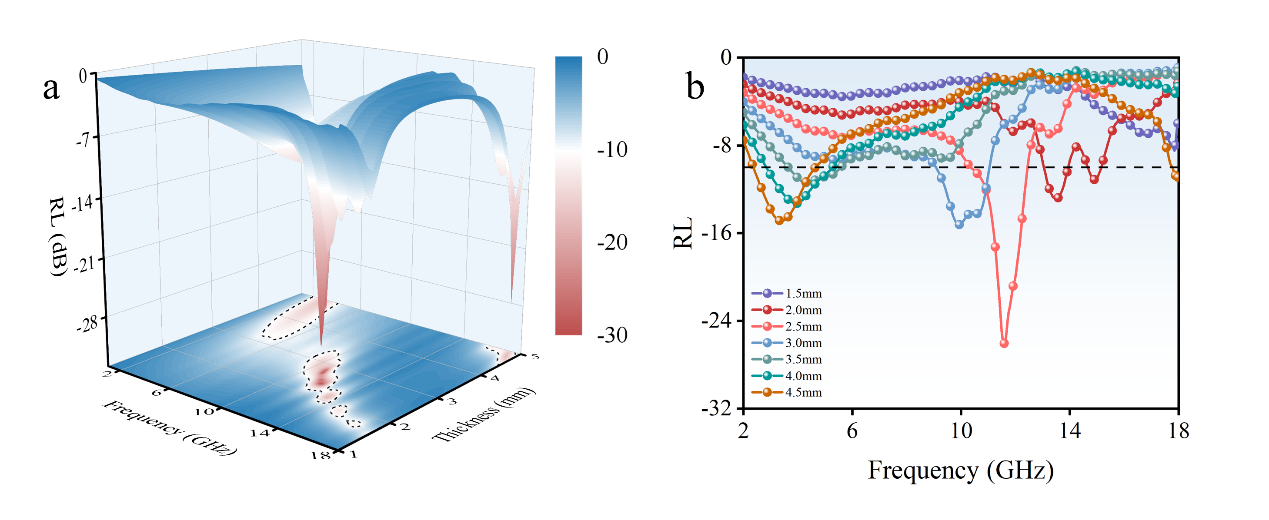


Figure S3 (a) 3D plots of RL as a function of frequency and absorber thickness for the sample with a hollow ratio of 0.0%. (b) RL–frequency curves at various matching thicknesses (1.5–4.5 mm) for the same sample (hollow ratio: 0.0%).


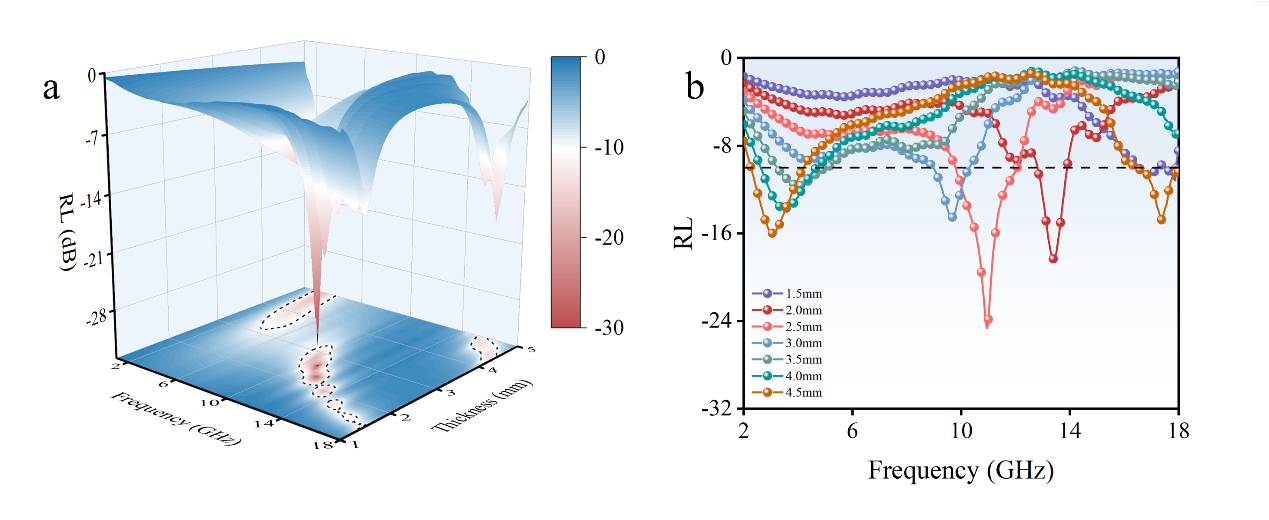


Figure S4 (a) 3D plots of RL as a function of frequency and absorber thickness for the sample with a hollow ratio of 17.1%. (b) RL–frequency curves at various matching thicknesses (1.5–4.5 mm) for the same sample (hollow ratio: 17.1%).
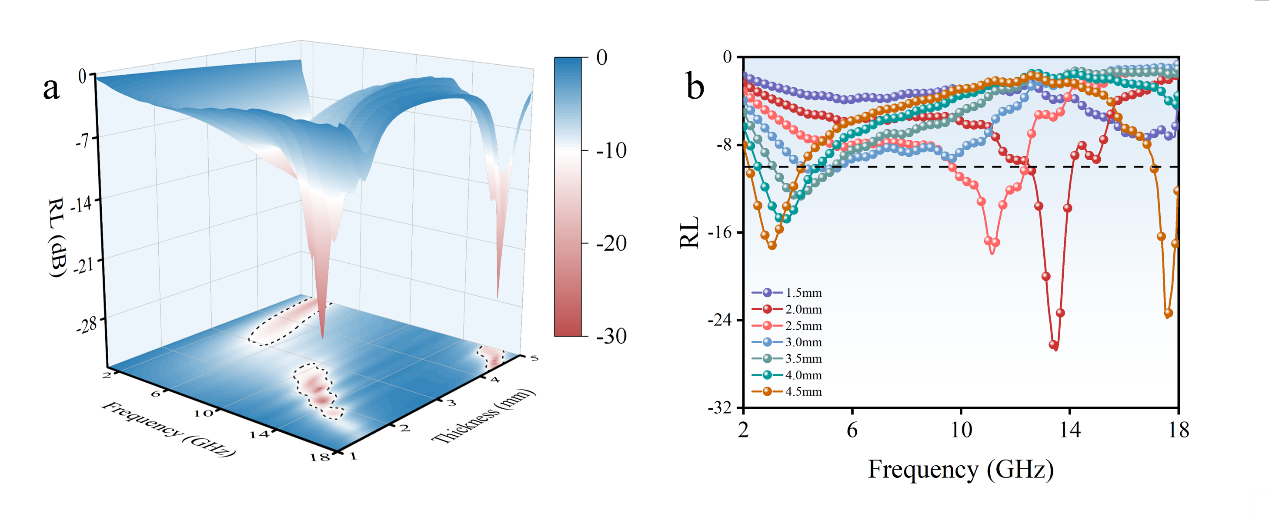


Figure S5 (a) 3D plots of RL as a function of frequency and absorber thickness for the sample with a hollow ratio of 32.2%. (b) RL–frequency curves at various matching thicknesses (1.5–4.5 mm) for the same sample (hollow ratio: 32.2%).


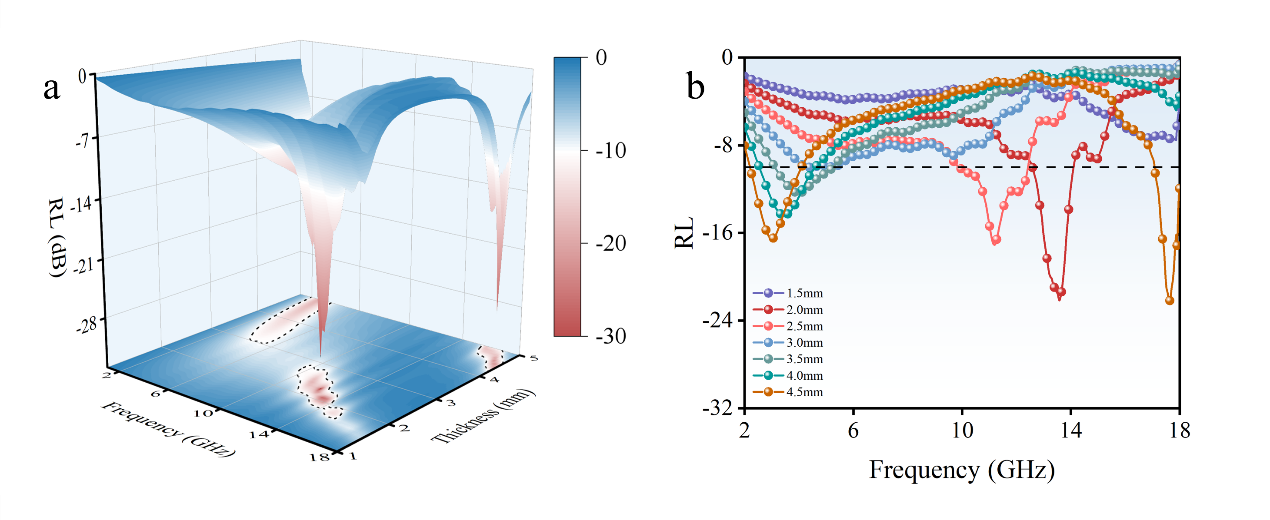


Figure S6 (a) 3D plots of RL as a function of frequency and absorber thickness for the sample with a hollow ratio of 46.6%. (b) RL–frequency curves at various matching thicknesses (1.5–4.5 mm) for the same sample (hollow ratio: 46.6%).


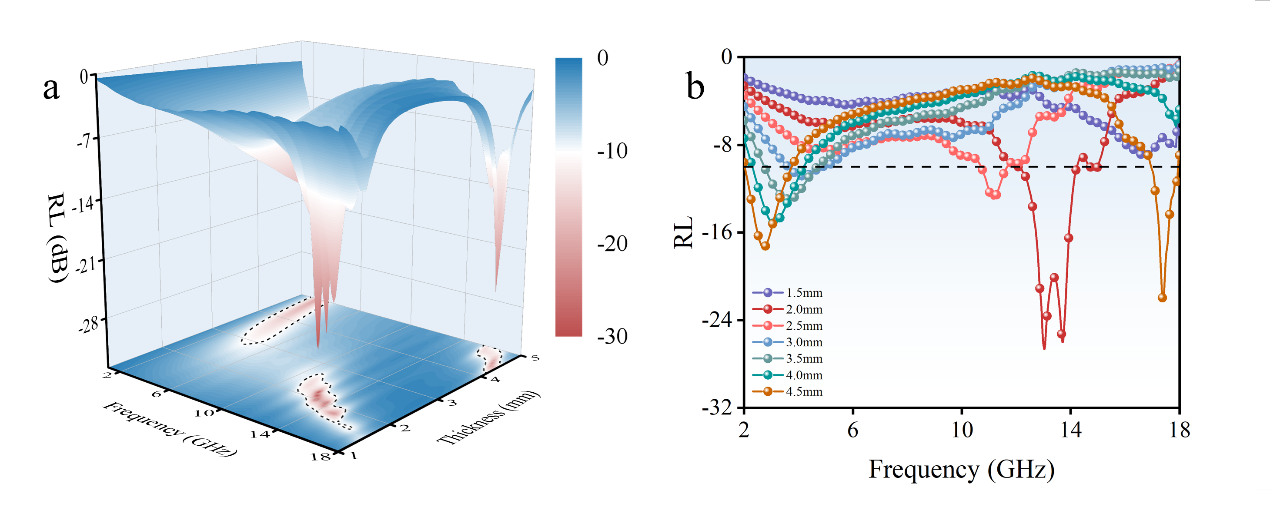


Figure S7 (a) 3D plots of RL as a function of frequency and absorber thickness for the sample with a hollow ratio of 54.1%. (b) RL–frequency curves at various matching thicknesses (1.5–4.5 mm) for the same sample (hollow ratio: 54.1%).


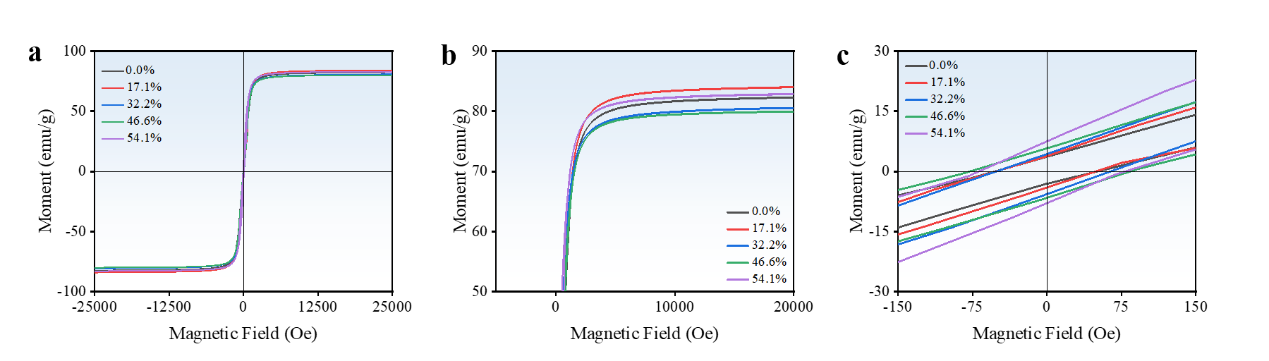


Figure S8 Magnetic hysteresis loops of Fe_3_O_4_ samples with different hollow rates measured at room temperature: (a) full-field hysteresis loops from −25,000 to 25,000 Oe; (b) magnified loops to observe Ms; c) magnified low-field region from −150 to 150 Oe to observe the coercivity.


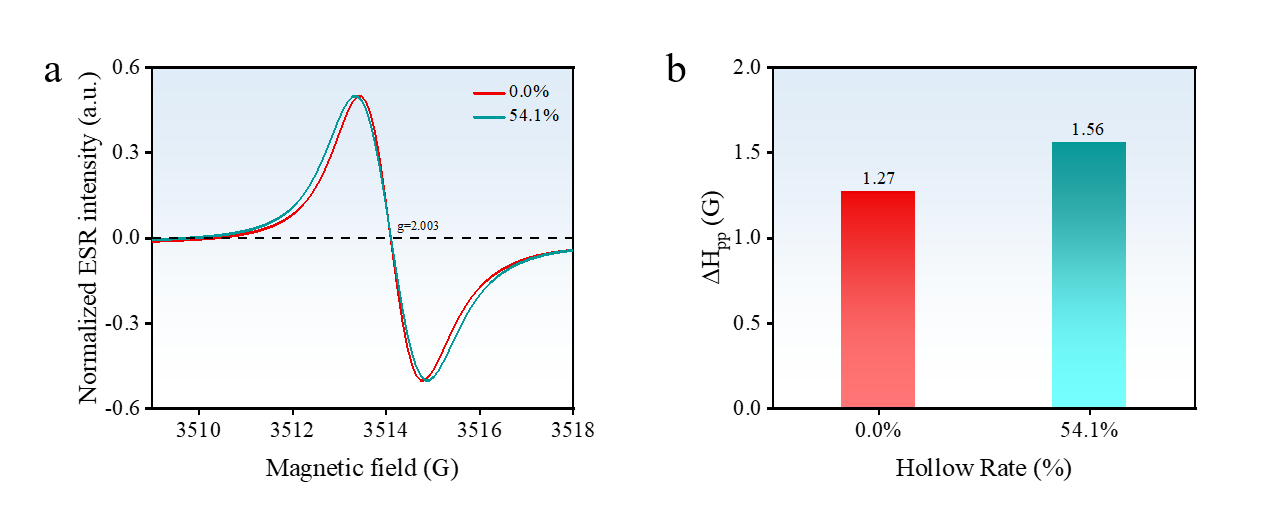


Figure S9. ESR spectra and linewidth analysis of Fe_3_O_4_ samples. (a) Peak-to-peak normalized ESR spectra of Fe_3_O_4_ samples with hollow rates of 0.0% and 54.1%. (b) The corresponding ESR peak-to-peak linewidth ΔH_pp_.


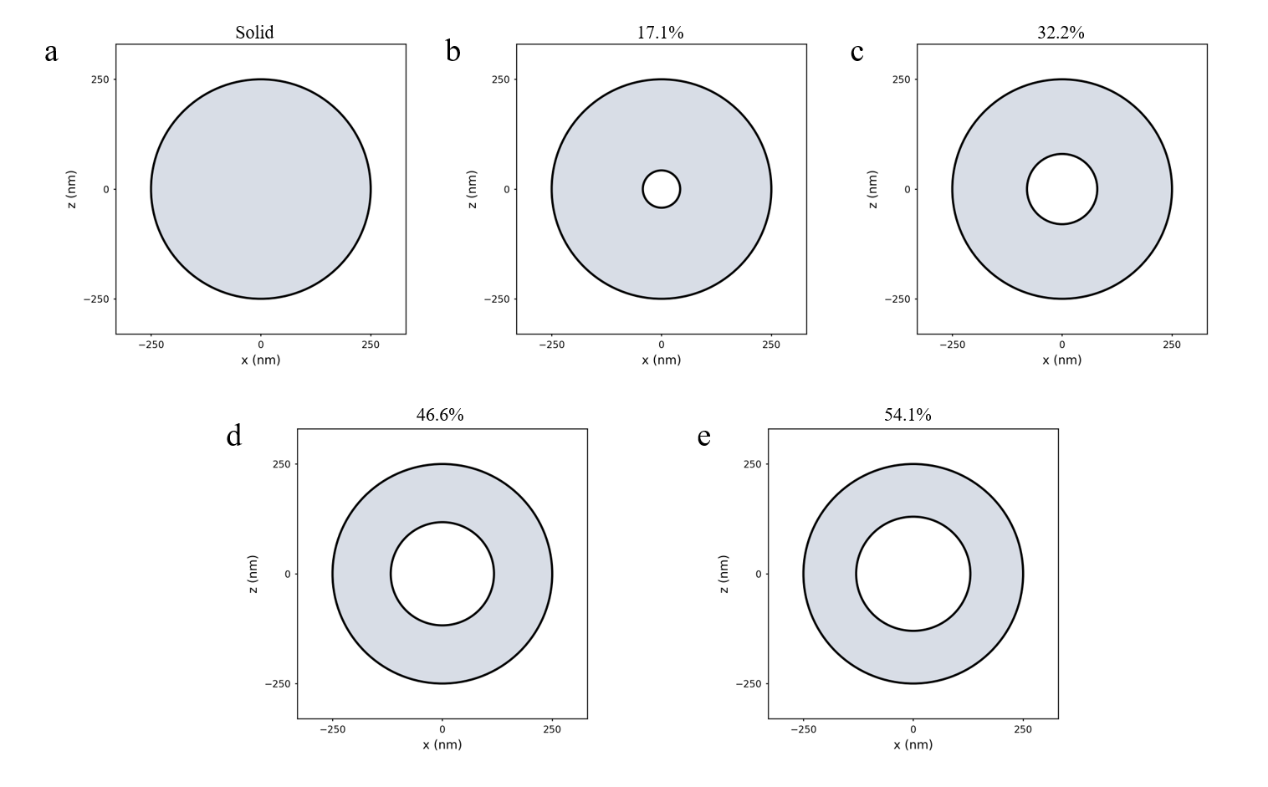


Figure S10. Cross-sectional geometrical models of Fe_3_O_4_ nanospheres used for COMSOL Multiphysics simulations. (a–e) Models with hollow rates of (a) 0.0% solid, (b) 17.1%, (c) 32.2%, (d) 46.6%, and (e) 54.1%, respectively.


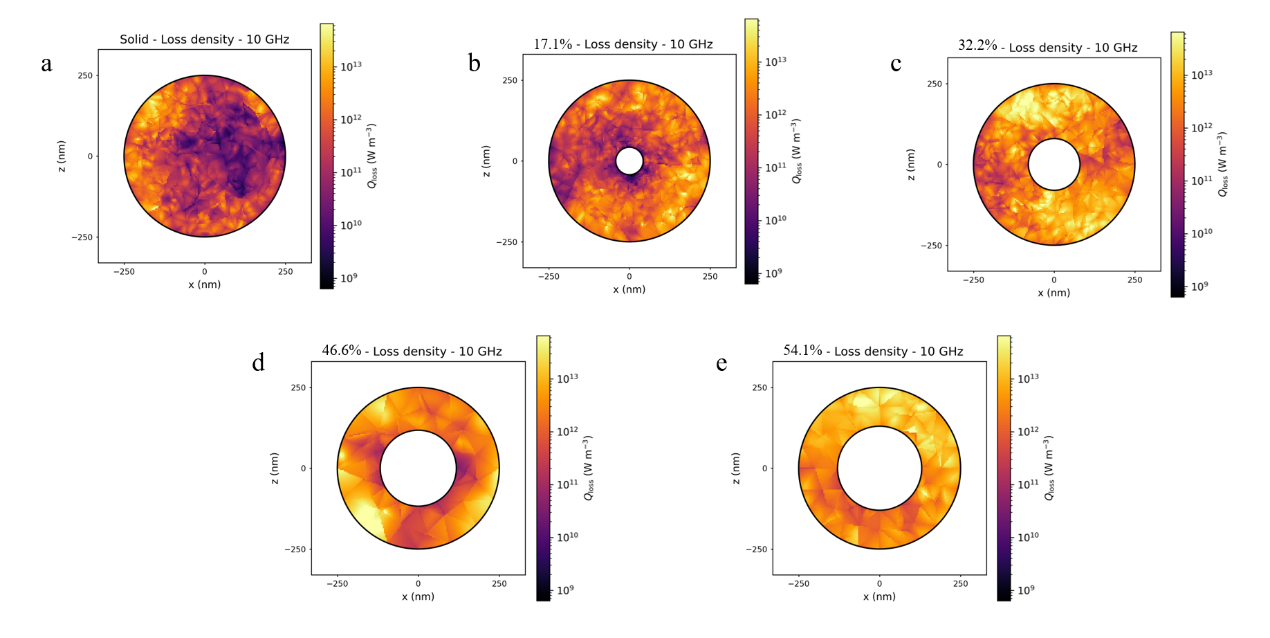


Figure S11. COMSOL Multiphysics -simulated electromagnetic loss-density distributions of Fe_3_O_4_ nanospheres at 10 GHz. (a–e) Loss-density distributions of Fe_3_O_4_ nanospheres with hollow rates of (a) 0.0% solid, (b) 17.1%, (c) 32.2%, (d) 46.6%, and (e) 54.1%, respectively.

**Table S1. Comparison of the EMA of this work with recently reported representative hollow magnetic absorbers.**

| **Material** | **EAB criterion** | **EAB range** | **Bandwidth** | **Thickness** | **Ref.** |
| --- | --- | --- | --- | --- | --- |
| Solid Fe_3_O_4_ | RL≤ −10 dB | 3.02-5.41 GHz | 2.39 GHz | 3.85 mm | This work |
| 54.1% hollow Fe_3_O_4_ | RL≤−10 dB | 2.69-4.68 GHz | 1.99 GHz | 3.50 mm | This work |
| MnFe_2_O_4_ nano-hollow spheres, 450 nm | RL≤−10 dB | 8.6–10.8 and 15.2–16.7 GHz | 3.70 GHz | 5.10 mm | [S1] |
| MnFO@CFO multilayered nano-hollow spheres | RL≤−10 dB | 5.20-7.40 GHz | 2.20 GHz | 4.50 mm | [S2] |
| MnFe_2_O_4_ nano-hollow spheres, 50 wt% | RL≤−10 dB | 15.80-19.40 GHz | 3.60 GHz | 4.20 mm | [S3] |

**Table S2. Comparison of low-frequency absorption features of this work with representative carbon-based and MXene-based absorbers.**

| **Material** | **EAB criterion** | **EAB range** | **Bandwidth** | **Thickness** | **Ref.** |
| --- | --- | --- | --- | --- | --- |
| Solid Fe_3_O_4_ | RL≤ −10 dB | 3.02-5.41 GHz | 2.39 GHz | 3.85 mm | This work |
| 54.1% hollow Fe_3_O_4_ | RL≤−10 dB | 2.69-4.68 GHz | 1.99 GHz | 3.50 mm | This work |
| N-doped hollow carbon capsules | RL≤ −10 dB | 12.7-17.9 GHz | 5.20 GHz | 2.00 mm | [S4] |
| Graphene aerogel/SiC nanowire/BN composite | RL≤−10 dB | 8.80-18.0 GHz | 9.20 GHz | 2.50 mm | [S5] |
| CNT film–Fe_3_O_4_–graphene composite | RL≤−10 dB | 12.0-16.7 GHz | 4.70 GHz | 1.50 mm | [S6] |
| Ti_3_C_2_ MXene nanosheets | RL≤ −10 dB | 11.20-18.0 GHz | 6.80 GHz | 2.00 mm | [S7] |
| Aligned 3D MXene/CNF cavities | RL≤−10 dB | 2.80-3.90 GHz | 1.10 GHz | 8.51 mm | [S8] |

**References**

[S1] D. Mandal, K. Mandal, "Enhancement of electromagnetic wave absorption in MnFe_2_O_4_ nano-hollow spheres," *Journal of Applied Physics* 129 (2021): https://doi.org/10.1063/5.0039560.

[S2] A. Gorai, D. Mandal, K. Mandal, "Multi-layered nano-hollow spheres for efficient electromagnetic wave absorption," *Nanotechnology* 32 (2021): https://doi.org/10.1088/1361-6528/ac020e.

[S3] D. Mandal, K. Mandal, "Electromagnetic wave attenuation properties of MFe_2_O_4_ (M = Mn, Fe, Co, Ni, Cu, Zn) nano-hollow spheres in search of an efficient microwave absorber," *Journal of Magnetism and Magnetic Materials* 536 (2021): https://doi.org/10.1016/j.jmmm.2021.168127.

[S4] J. Chen, P. Miao, E. E. Lin, T. Bai, S. K. Smoukov, J. Kong, "Enhanced microwave absorption performance of light weight N-doped carbon nanoparticles Electronic supplementary information " *RSC Advances* 11 (2021): 7954-7960, https://doi.org/10.1039/d0ra08455g.

[S5] X. You, H. Ouyang, R. Deng, Q. Zhang, Z. Xing, X. Chen, et al., "Graphene Aerogel Composites with Self-Organized Nanowires-Packed Honeycomb Structure for Highly Efficient Electromagnetic Wave Absorption," *Nano-Micro Letters* 17 (2024): 47, https://doi.org/10.1007/s40820-024-01541-y.

[S6] J. S. Li, W. B. Lu, J. Suhr, H. Chen, J. Q. Xiao, T. W. Chou, "Superb electromagnetic wave-absorbing composites based on large-scale graphene and carbon nanotube films," *Scientific Reports* 7 (2017): https://doi.org/10.1038/s41598-017-02639-7.

[S7] W. L. Feng, H. Luo, Y. Wang, S. F. Zeng, L. W. Deng, X. S. Zhou, et al., "Ti_3_C_2_ MXene: a promising microwave absorbing material," *RSC Advances* 8 (2018): 2398-2403, https://doi.org/10.1039/c7ra12616f.

[S8] B. Shan, Y. Wang, X. Ji, Y. Huang, "Enhancing Low-Frequency Microwave Absorption Through Structural Polarization Modulation of MXenes," *Nano-Micro Letters* 16 (2024): 212, https://doi.org/10.1007/s40820-024-01437-x.
